# Impact of strawberry consumption on blood pressure in adults: GRADE-assessed systematic review and dose-response meta-analysis of data from randomized controlled trials

**DOI:** 10.22038/ajp.2024.25222

**Published:** 2025

**Authors:** Mostafa Shahraki Jazinaki, Mohammad Safarian, Mohammad Rashidmayvan, Seyyed Mostafa Arabi, Amirhossein Sahebkar

**Affiliations:** 1 *Department of Nutrition, Faculty of Medicine, Mashhad University of Medical Sciences, Mashhad, Iran*; 2 *Metabolic Syndrome Research Center, Mashhad University of Medical Sciences, Mashhad, Iran*; 3 *Department of Nutrition, Food Sciences and Clinical Biochemistry, School of Medicine, Social Determinants of Health Research Center, Gonabad University of Medical Science, Gonabad, Iran*; 4 *Noncommunicable Diseases Research Center, Neyshabur University of Medical Sciences, Neyshabur, Iran*; 5 *Healthy Ageing Research Center, Neyshabur University of Medical Sciences, Neyshabur, Iran*; 6 *Applied Biomedical Research Center, Basic Sciences Research Institute, Mashhad University of Medical Sciences, Mashhad, Iran*; 7 *Centre for Research Impact & Outcome, Chitkara College of Pharmacy, Chitkara University, Rajpura, Punjab 140401, India*; 8 *Biotechnology Research Center, Pharmaceutical Technology Institute, Mashhad University of Medical Sciences, Mashhad, Iran*

**Keywords:** Strawberry, Fragaria x ananassa, Systolic blood pressure, Diastolic blood pressure Systematic review, Meta-analysis

## Abstract

**Objective:**

This systematic review and meta-analysis aimed to assess the impact of strawberry (*Fragaria x ananassa*) consumption on systolic (SBP) and diastolic blood pressure (DBP).

**Materials and Methods:**

PubMed, Web of Science, Scopus, and Google Scholar were searched to find relevant randomized controlled trials (RCTs). Meta-analysis was carried out by using the random effect model, and the I^2^ index was used to assess heterogeneity among included trials.

**Results:**

Out of the 81 studies obtained, eight were eligible to be included in this review. The pooled effect size of 12 effect sizes indicated that strawberry consumption had no significant effect on SBP (WMD: 0.96 mmHg, 95% CI -0.26 to 2.20, p = 0.12), or DBP levels (WMD: -0.33 mmHg, 95% CI -1.31 to 0.65, p = 0.50). Subgroup analysis showed that consumption of freeze-dried strawberry powder at a dose of ≤ 25 g/day or strawberry intake in people under the age of 50 significantly increased SBP levels. Also, strawberry intake in individuals aged 50 or older led to a significant decrease in DBP levels.

**Conclusion:**

This review suggests that strawberry consumption may not be an effective strategy for hypertension management. However, more RCTs are needed to draw a definite conclusion.

## Introduction

Hypertension (HTN), also known as the silent killer, remains one of the major global health challenges and is the cause of 9.4 million deaths annually (Kearney et al., 2005; Campbell et al., 2014; Norouzy et al., 2017). High blood pressure is defined as a condition in which the blood pressure rises to over 140/90 mmHg (Kearney et al., 2005). HTN is a known risk factor for cardiovascular diseases (CVDs) and renal failure. Also, it is the leading cause of half of the deaths related to CVDs, type 2 diabetes (T2D), and chronic kidney disease (CKD) (Kearney et al., 2005; Castro et al., 2015). By 2025, more than 500 million individuals globally will be impacted by HTN (Islam et al., 2015). High blood pressure as a clinical syndrome is caused by multifaceted causes related to genetics, the environment, and their interactions (Abed and Abu-Haddaf, 2013). Some of the risk factors for high blood pressure are modifiable, such as physical activity (Avila-Palencia et al., 2019), obesity (Elmer et al., 2006), alcohol consumption (Xin et al., 2001), and nutrition-related factors (Ghanbari et al., 2022). Small reductions in blood pressure can have many health benefits and reduce the burden of CVDs, so a 10 mm Hg reduction in SBP significantly reduces the risk of cardiovascular events (Ettehad et al., 2016). Therefore, blood pressure management is of clinical interest (Ettehad et al., 2016). Lifestyle changes such as exercise, reducing sodium intake, and drug therapy are the most common treatments for hypertension (Al Shukor et al., 2013). Dietary factors play an undeniable role in preventing and developing hypertension, and interventional nutritional modifications can contribute to the management of these patients (Eslampour et al., 2020). Furthermore, patients usually prefer healthy eating and exercise to drug consumption due to the lack of side effects of medications (Setayesh et al., 2021). The Dietary Approaches to Stop Hypertension (DASH) is one of the main strategies for preventing or managing hypertension (Filippou et al., 2020). One of the suggestions of the DASH eating plan is to increase fruit and vegetable intake (Campbell, 2017). Previous studies have reported a link between consuming fruits and vegetables rich in polyphenols and reducing the mortality rate from CVDs (Hertog et al., 1995; Peterson et al., 2012). Additionally, consuming some anthocyanin-rich plants has significantly reduced the risk of hypertension (Cassidy et al., 2011). Strawberries (*Fragaria* x *ananassa*) are a native product of North America (Giampieri et al., 2012), and the fifth most consumed fruit in the USA (Feresin et al., 2017). Strawberries are rich in nutrients such as folic acid, vitamin C, and manganese (Giampieri et al., 2012). They also contain non-nutritive compounds such as polyphenols, flavonoids (flavonols and anthocyanins), and hydrolyzable tannin (Giampieri et al., 2012), which gave strawberries potent anti-inflammatory (Parelman et al., 2012; Liu and Lin, 2012; Liu and Lin, 2013), and antioxidant properties (Wang et al., 1996; Alvarez-Suarez et al., 2014). According to the known link between inflammation, oxidative stress, and increased blood pressure, the hypothesis is that strawberry consumption may significantly reduce blood pressure (Zhang et al., 2022). The results of RCTs investigating the effects of strawberry consumption on blood pressure are conflicting. This systematic review and meta-analysis on RCTs aimed to investigate the impact of strawberries on blood pressure in adults.

## Materials and Methods

This systematic review and meta-analysis was carried out according to the updated Preferred Reporting Items for Systematic Reviews and Meta-Analyses (PRISMA) protocol (Page et al., 2021), and its protocol was registered in PROSPERO database with the registration ID: CRD42023450856.

### Search strategy

The search strategy, which included MeSH and non-MeSH keywords, was carried out without time or language restrictions in PubMed, Scopus, and Web of Science databases until May 2024. The following search terms were used: ("Strawberry" OR "Fragaria x ananassa") AND ("Blood pressure" OR "Systolic blood pressure" OR "SBP" OR "Diastolic blood pressure" OR "DBP") AND (“Randomized controlled trials" OR “RCT”). The reference lists of all eligible trials were checked to minimize the risk of missing articles with inclusion criteria, and Google Scholar was searched manually. Details of the search strategy in each of the databases are provided in Supplementary Table 1.

### Eligibility criteria

Based on the inclusion criteria for this review, two researchers (M.Sh.J) and (S.M.A) independently screened the obtained papers based on their titles and abstracts. The inclusion criteria for this review were designed by the PICOS framework (O'Connor et al., 2008; Amir-Behghadami and Janati, 2020): (P (Population): Adults (≥18 years old), I (intervention): strawberries intake, C (comparison): control group, S (type of study): RCTs).

### Data extraction

Two researchers (S.M.A) and (M.R.M) independently extracted the relevant data from each included article, including the first author's name, country, year of publication, number of participants in each group, mean age and BMI of participants in each group, type and dosage of intervention (g/day), type of control, the duration of the intervention and the mean changes and SDs in systolic and diastolic blood pressure in each group during the intervention. Disagreements were discussed until a mutual understanding was achieved.

### Risk of bias assessment

The quality assessment of included studies was done by two researchers (S.M.A) and (M.Sh.J) independently, using Cochrane's risk-of-bias assessment tool (ROB 1) (Higgins and Green, 2010). This tool evaluates the risk of bias in the following seven domains: Random sequence generation, allocation concealment, blinding of participants and personnel, blinding of outcome assessment, incomplete outcome data, selective reporting, and other sources of bias. The risk of bias in each domain was classified as low, unclear, or high. In addition, the general risk of bias for each included trial was categorized into three levels: low, moderate, and high.

### Statistical analysis

The overall effect sizes are reported as Weighted mean differences (WMD) and 95% confidence interval (CI). Overall effect sizes were estimated using the random-effects model by DerSimonian and Laird (DerSimonian and Laird, 1986). When studies have reported SEM instead of SDs, the following formula was used to calculate SD: SD = SEM * √n. In this formula, n is the number of participants in each group (Hozo et al., 2005). If the mean changes were not mentioned, it was determined by subtracting values in the baseline from the values at the end of the intervention (Bahari et al., 2024). Also, the following formula was used to estimate SD changes: 

SD change = square root [(SD baseline) ^2^ + (SD final) ^2^ − (2 * R * SD baseline * SD final) (Borenstein et al., 2021, Jazinaki et al., 2024).

The heterogeneity among the included trials was evaluated using Cochrane's Q test and I^2^ index (I^2^ > 40% or p-value < 0.05 indicated significant heterogeneity) (Higgins et al., 2003). To find potential sources of heterogeneity, subgroup analysis was carried out based on the following predetermined criteria: country (Non-USA, and USA), study design, trial duration (≥8, and <8 weeks), intervention type (Fresh strawberries fruit, and freeze-dried strawberry powder), freeze-dried strawberry powder dosage (g/day) (>25, and ≤25 g/d), gender (Both sexes, and only female), Age (<50, and ≥50 years), and Baseline BMI (overweight, and obesity). A sensitivity analysis examined each study's influence on the overall effect size (Sahebkar, 2014). Publication bias was assessed through Egger regression, Begg correlation, and visual examination of funnel plot graphs (Egger et al., 1997, Begg, 1994). A meta-regression analysis was conducted to evaluate the linear relationship between the dose and duration of freeze-dried strawberry powder consumption and outcome change (SBP and DBP) (Xu and Doi, 2018). Fractional polynomial modeling was performed to evaluate the non-linear relationship between features of freeze-dried strawberry powder intake (dose and duration) and changes in outcome levels.

### Certainty assessment

The certainty of the evidence in this review was assessed using the GRADE framework (Guyatt et al., 2008). The quality of evidence was evaluated in the following five subclasses: Risk of bias (Guyatt et al., 2011e), Inconsistency (Guyatt et al., 2011c), Indirectness (Guyatt et al., 2011b), Imprecision (Guyatt et al., 2011a), and publication bias (Guyatt et al., 2011d). The overall quality of evidence for each outcome was categorized into four levels: very low, low, moderate, and high. 

## Results

**Figure 1:**
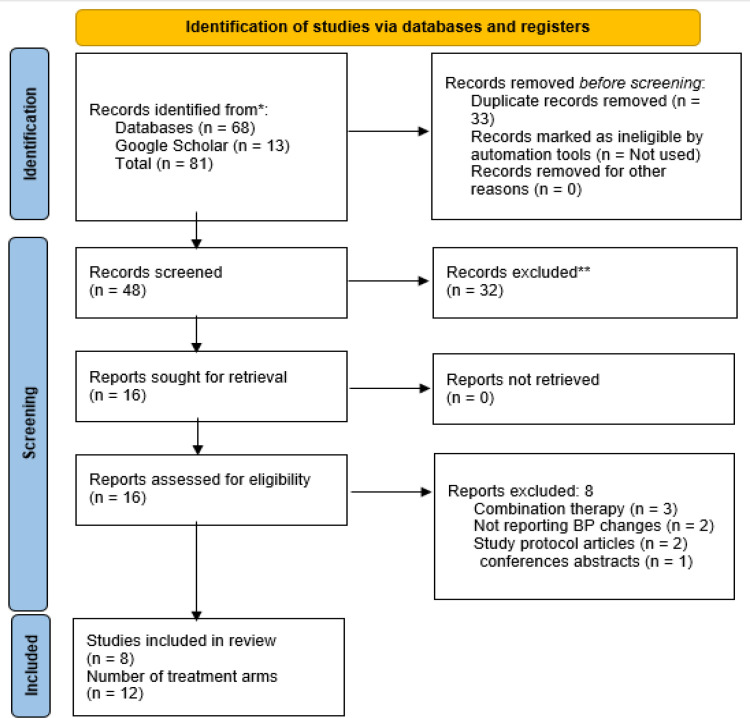
Flowchart of study selection for inclusion in the meta-analysis

A total of 81 studies were found in the initial comprehensive search (Databases: 68 and Google Scholar: 13). After identifying and removing 33 duplicate studies, 48 were screened based on their title and abstract; as a result, 32 studies were excluded. Furthermore, reading the full text was necessary for the 16 remaining articles to assess the eligibility criteria. Following that, eight studies were excluded: 3 due to combination therapy, 2 for being a study protocol article, 1 for being only a conference abstract, and 2 due to not reporting the relevant outcomes. Finally, eight studies (with 12 arms) were eligible for inclusion in this systematic review and meta-analysis (Figure 1) (Jenkins et al., 2008; Basu et al., 2010; Basu et al., 2014; Amani et al., 2014; Feresin et al., 2017; Huang et al., 2021; Basu et al., 2021; Richter et al., 2023). 

### Findings from the systematic review

**Figure 2:**
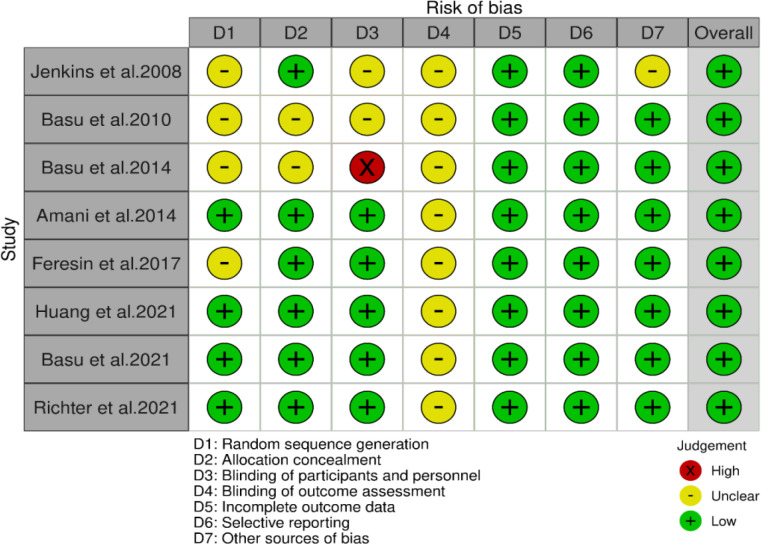
Risk of bias assessment traffic light plot

The characteristics of the included trials are summarized in Table 1. The countries where the eligible trials were carried out included the USA (Basu et al., 2010; Basu et al., 2014; Feresin et al., 2017; Huang et al., 2021; Basu et al., 2021; Richter et al., 2023), Canada (Jenkins et al., 2008), and Iran (Amani et al., 2014). Among the included studies, 4 had parallel design (Basu et al., 2010; Basu et al., 2014; Amani et al., 2014; Feresin et al., 2017), and 4 were crossover (Jenkins et al., 2008; Huang et al., 2021; Basu et al., 2021; Richter et al., 2023). The mean age of the participants ranged from 46.5 (Basu et al., 2010), to 62 years (Jenkins et al., 2008), and their mean BMI was between 26.5 (Jenkins et al., 2008), and 37.7 Kg/m^2 ^(Basu et al., 2010). Seven studies were conducted on both sexes (Jenkins et al., 2008; Basu et al., 2010; Basu et al., 2014; Amani et al., 2014; Huang et al., 2021; Basu et al., 2021; Richter et al., 2023), and one on only females (Feresin et al., 2017). The studied populations included individuals with hyperlipidemia (Jenkins et al., 2008), metabolic syndrome (Basu et al., 2010), type 2 diabetes (Amani et al., 2014), moderate hypercholesterolemia (Huang et al., 2021), obesity along with elevated serum LDL-C levels (Basu et al., 2021), obesity along with elevated cholesterol levels (Richter et al., 2023), abdominal adiposity along with elevated serum lipids (Basu et al., 2014), and postmenopausal women with pre- and stage 1- hypertension (Feresin et al., 2017). The intervention in all included studies was performed using freeze-dried strawberry powder, except for one study that used fresh strawberries (Jenkins et al., 2008). In the included trials, the intervention's length varied from 4 (Jenkins et al., 2008; Huang et al., 2021; Basu et al., 2021; Richter et al., 2023), to 12 weeks (Basu et al., 2014). According to the risk of bias assessment of the included studies that was conducted based on the ROB 1 tool, the general risk of bias for all of the eligible trials was identified as low, which means all of them had less than two high risk of bias items in the seven domains of the ROB 1. Also, all of the included trials had a good overall quality (Details of the assessment of the risk of bass in each subclass are shown in Figure 2 and Supplementary Figure 1 and Supplementary Table 2) (Jenkins et al., 2008; Basu et al., 2010; Basu et al., 2014; Amani et al., 2014; Huang et al., 2021; Basu et al., 2021; Richter et al., 2023; Feresin et al., 2017). 

**Table 1:** Characteristic of the studies included in the meta-analysis

**studies**	**Country**	**Study Design**	**Participant**	**Sample size and Sex**	**Sample size**		**Trial Duration** **(Week)**	**Means Age**		**Means BMI**		**Intervention**	
					**IG**	**CG**		**IG**	**CG**	**IG**	**CG**	**Strawberries dose (g/d)**	**Control group**
Jenkins et al.2008	Canada	Crossover, R, C	Hyperlipidemia	28 B	28	28	4 (30 days)	62±5.29	62±5.29	26.5±3.17	26.5±3.17	Strawberries 452 g/d	Oat bran bread
Basu et al.2010	USA	Parallel, R, C	Metabolic syndrome	27 B	15	12	8	48.0±20.52	45.0±10.39	39.0±7.74	36.4±10.39	FDS powder beverage 50 g/d ∼500 g fresh fruit	Water
Basu et al.2014(a)	USA	Parallel, R, C	Abdominal Adiposity and Elevated Serum Lipids	30 B	15	15	12	50.0±10	48±10	34.5±4.4	37.0±4.4	FDS powder beverage 25 g/d ∼250 g fresh fruit	Calorie- and fiber-matched control beverage
Basu et al.2014(b)	USA	Parallel, R, C	Abdominal Adiposity and Elevated Serum Lipids	30 B	15	15	12	49±11	48±10	38.0±7.1	35.0±5.2	FDS powder beverage 50 g/d ∼500 g fresh fruit	Calorie- and fiber-matched control beverage
Amani et al.2014	Iran	Parallel, R, PC, DB	Type 2 diabetes	36 B	19	17	6	51.9±8.2	51.1±13.8	27.36±4.23	28.58±4.7	FDS powder beverage 50 g/d ∼500 g fresh fruit	Color- and fiber-matched Placebo beverage
Feresin et al.2017(a)	USA	Parallel, R, PC, DB	Pre- and Stage 1- Hypertensive Postmenopausal Women	40 F	20	20	8	61±4.47	58±4.47	31.0±4.47	32.1±3.13	FDS powder 25 g/d ∼1.5 cups of sliced fresh strawberries	Placebo
Feresin et al.2017(b)	USA	Parallel, R, PC, DB	Pre- and Stage 1- Hypertensive Postmenopausal Women	40 F	20	20	8	59±4.47	58±4.47	32.7±4.91	32.1±3.13	FDS powder 50 g/d ∼3 cups of sliced fresh strawberries	Placebo
Huang et al.2021	USA	Cross over, R, PC, DB	Moderate Hypercholesterolemia	34 B	34	34	4	53±5.83	53±5.83	30.6±3.49	30.6±3.49	FDS powder beverage 50 g/d ∼500 g fresh strawberries	Energy and volume-matched control beverage
Basu et al.2021(a)	USA	Cross over, R, PC, DB	Obesity and Elevated Serum LDL Cholesterol	33 B	33	33	4	53 ± 13	53 ± 13	33 ± 3	33 ± 3	FDS powder 13 g/d ∼1.0 serving of fresh strawberries	Control powder
Basu et al.2021(b)	USA	Cross over, R, PC, DB	Obesity and Elevated Serum LDL Cholesterol	33 B	33	33	4	53 ± 13	53 ± 13	33 ± 3	33 ± 3	FDS powder 32 g/d ∼2.5 serving of fresh strawberries	Control powder
Richter et al.2023(a)	USA	Cross over, R, PC, DB	Overweight or Obesity and Elevated Cholesterol	40 B	40	40	4	49.5±9.2	49.5±9.2	29.4±2.7	29.4±2.7	FDS powder 13 g/d ∼1.0 serving of fresh strawberries	Placebo
Richter et al.2023(b)	USA	Cross over, R, PC, DB	Overweight or Obesity and Elevated Cholesterol	40 B	40	40	4	49.5±9.2	49.5±9.2	29.4±2.7	29.4±2.7	FDS powder 40 g/d∼3.0 serving of fresh strawberries	Placebo

Abbreviations: RCT, randomized clinical trial; R, randomized; C, Control; P, Placebo; DB, Double-blind; B, Both; F, Female; M, Male; Int, Intervention; C, Control; IG, Intervention group; CG, control group; DB, Double-Blind; NR, Not Reported; BMI Body mass index; USA, The United States of America; LDL, low-density lipoproteins; FDS, freeze-dried strawberry

### Findings from the meta-analysis

#### The effect of strawberry consumption on SBP

**Figure 3:**
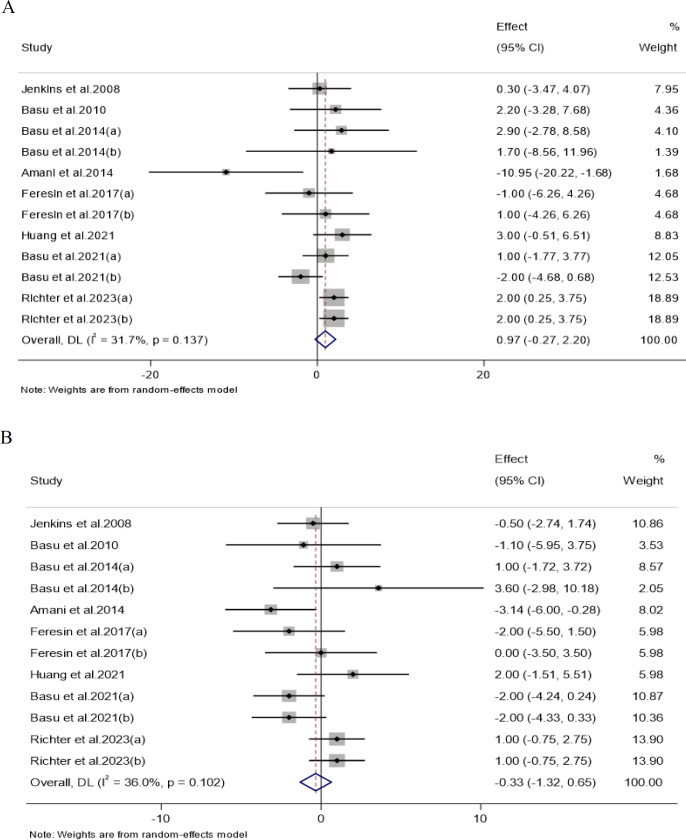
Forest plot detailing weighted mean difference and 95% confidence intervals (CIs) for the effects of strawberry consumption on (A) SBP (mmHg); and (B) DBP (mmHg)

After combining 12 effect sizes from 8 studies, it was found that strawberry intake did not significantly alter the SBP levels compared to the control groups (WMD: 0.96 mmHg, 95% CI -0.26 to 2.20, p = 0.12). Furthermore, no significant heterogeneity was seen among the trials that were included (I^2^: 31.7%, p =0.13) (Figure 3A). Subgroup analysis showed that strawberry intake in individuals aged less than 50 years or freeze-dried strawberry powder consumption with a dosage of ≤ 25 g/day led to a significant increase in SBP levels. Also, this analysis detected a significant rise in SBP levels followed by strawberry intake in the studies conducted in the USA (Table 2). The effect of strawberry consumption on DBP Pooling of 12 effect sizes from 8 studies showed that strawberry consumption did not lead to significant changes in DBP compared to control groups (WMD: -0.33 mmHg, 95% CI -1.31 to 0.65, p = 0.50). 

Also, there was no significant heterogeneity between the pooled studies (I2: 36.0%, p =0.10) (Figure 3B). Furthermore, subgroup analysis demonstrated that strawberry intake could significantly decrease DBP levels in individuals aged 50 or older (Table 2).

### Meta-regression and dose-response analyses

**Figure 4:**
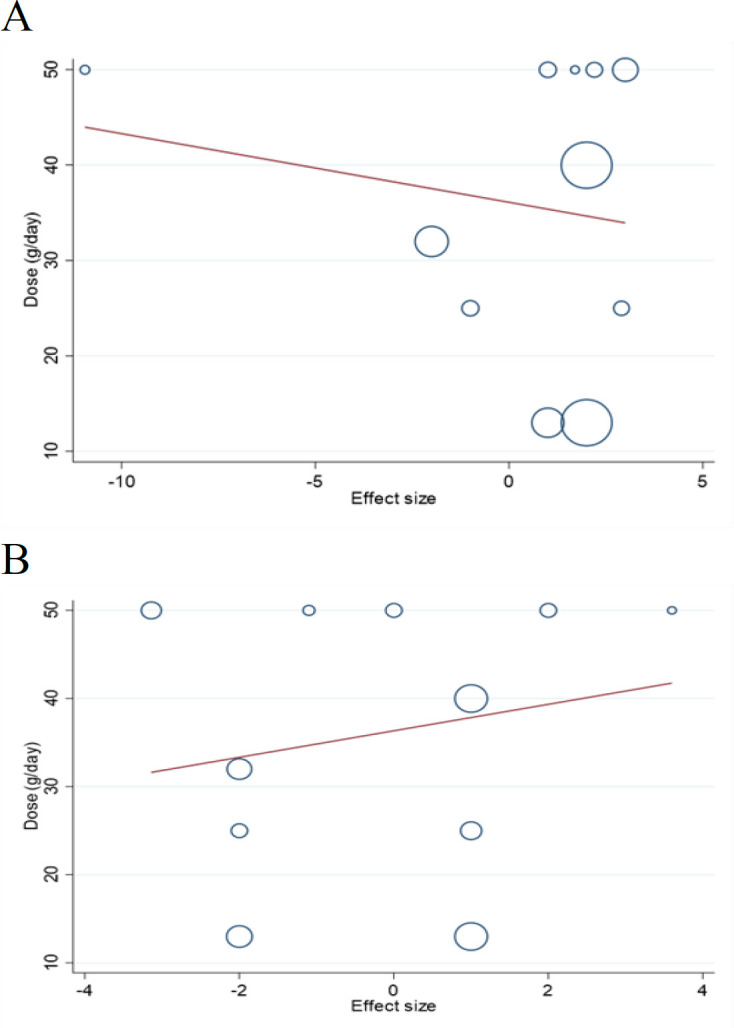
Linear dose-response relations between the dosage of strawberry (freeze-dried strawberry powder) consumption (g/day) and absolute mean differences in (A) SBP (mmHg) and (B) DBP (mmHg)

**Figure 5:**
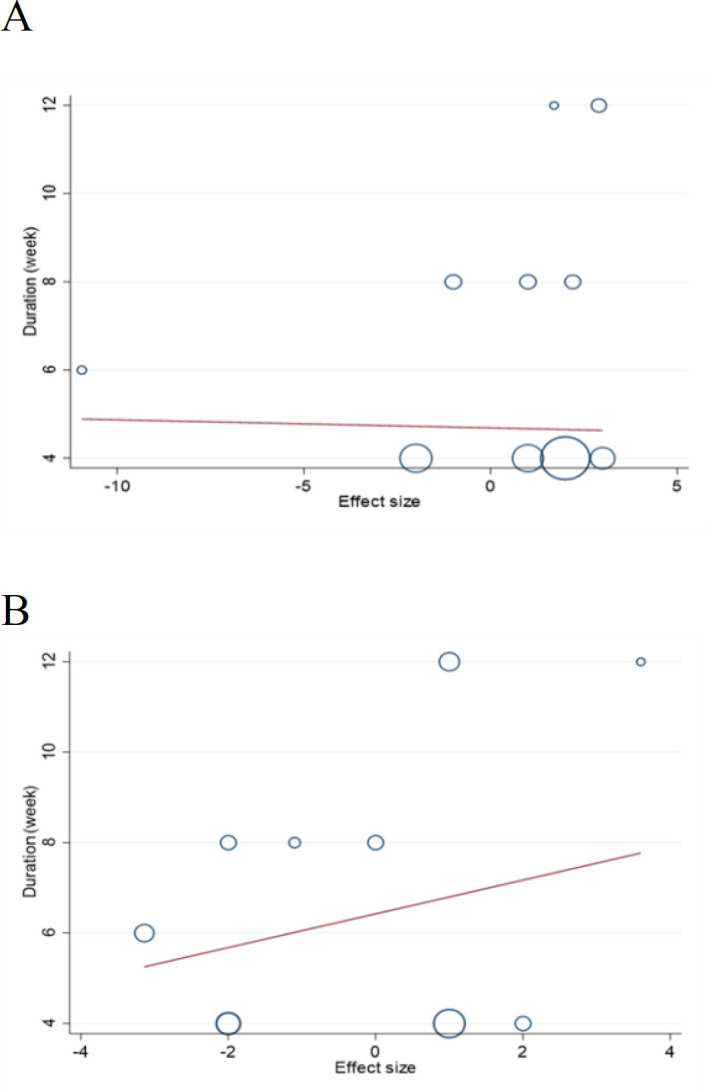
Linear dose-response relations between duration of strawberry (freeze-dried strawberry powder) consumption and absolute mean differences in (A) SBP (mmHg) and (B) DBP (mmHg)

**Figure 6:**
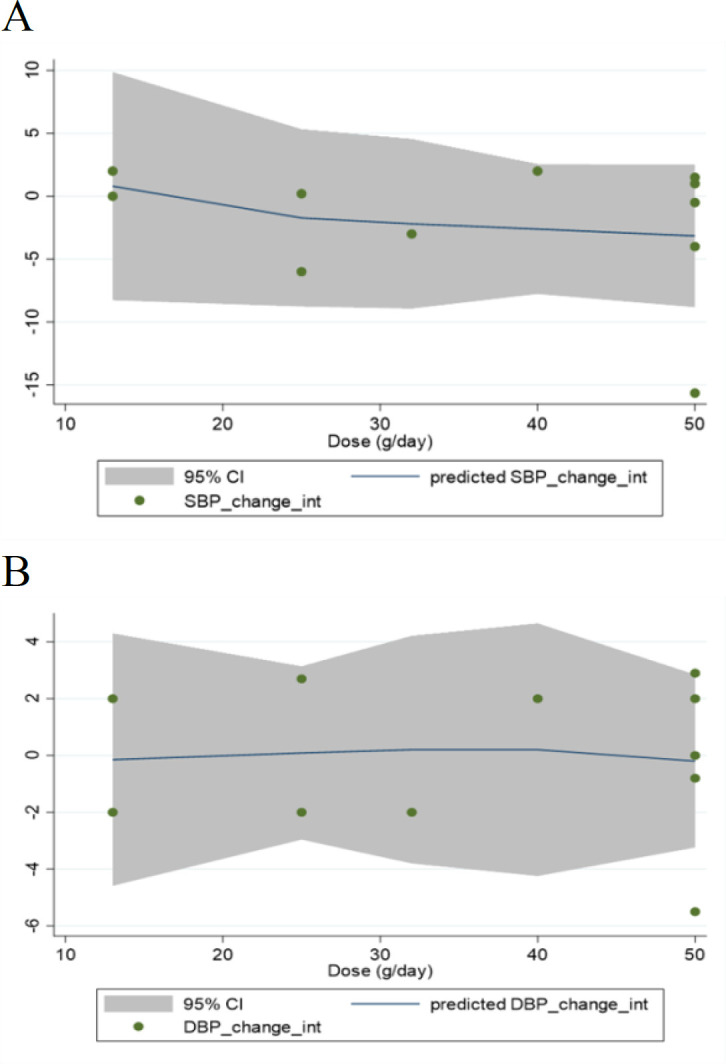
Non-linear dose-response relations between the dosage of strawberry (freeze-dried strawberry powder) consumption (g/day) and absolute mean differences in (A) SBP (mmHg) and (B) DBP (mmHg).

**Figure 7:**
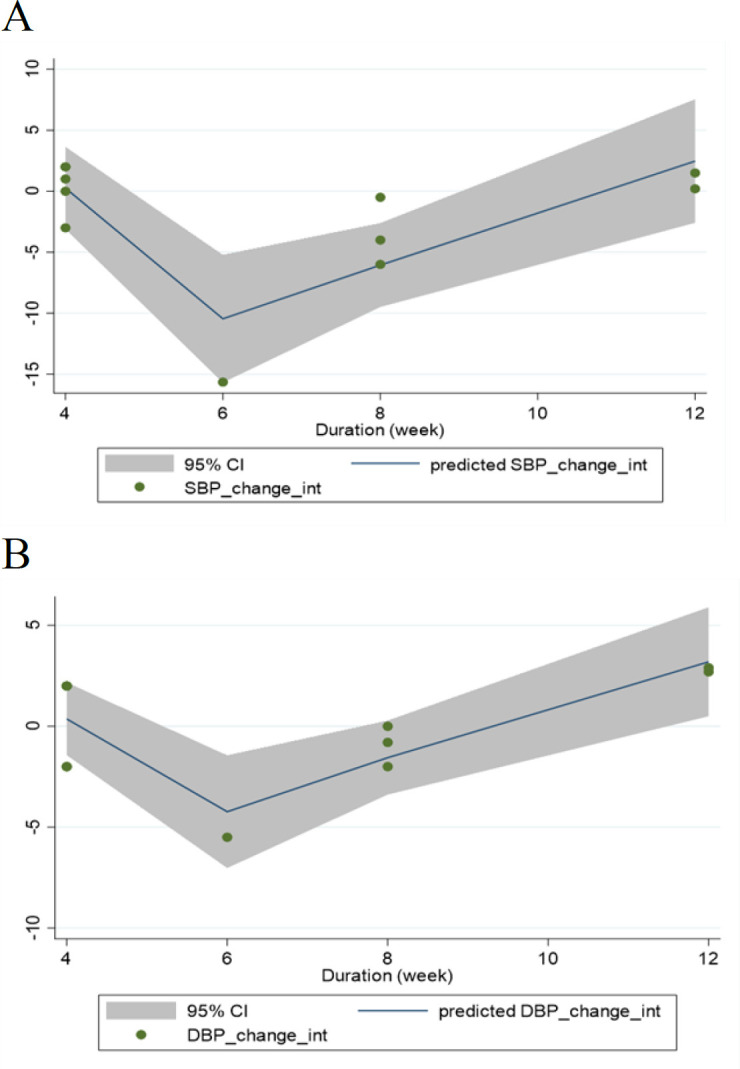
Non-linear dose-response relations between duration of strawberry (freeze-dried strawberry powder) consumption and absolute mean differences in (A) SBP (mmHg) and (B) DBP (mmHg)

Meta-regression did not report a significant linear relationship between the dosage of freeze-dried strawberry powder intake and blood pressure level changes (SBP: coefficients = -0.71, P _linearity_ = 0.58; Figure 4A, DBP: coefficients = 1.50, P _linearity_ = 0.54; Figure 4B). Also, no significant linear relationship was detected between the duration of freeze-dried strawberry powder consumption and outcome changes (SBP: coefficients = -0.01, P _linearity_ = 0.95; Figure 5A, DBP: coefficients = 0.37, P _linearity_ = 0.48; Figure 5B). Fractional polynomial modeling revealed a significant non-linear relationship between the duration of freeze-dried strawberry powder consumption and outcomes changes (SBP: coefficients = -1861.77, P _non-linearity_ = 0.004; Figure 7A, DBP: coefficients = -980.49, P _non-linearity_ = 0.005; Figure 7B). Furthermore, it seems that the intervention duration of 6 weeks for freeze-dried strawberry powder intake led to a more significant reduction of SBP and DBP than other durations applied in the included trials. However, there was no significant non-linear relationship between the dosage of freeze-dried strawberry powder consumption and blood pressure changes (SBP: coefficients = -0.006, P _non-linearity_ = 0.88; Figure 6A, DBP: coefficients = -0.02, P _non-linearity_ = 0.88; Figure 6B).

**Table 2:** Description of subgroup analyses of strawberry consumption on blood pressure.

	**NO**	**WMD (95%CI)**	**P-value**	**heterogeneity**		
				**P heterogeneity**	**I** ^2^	**P between sub-groups**
**Subgroup analyses of strawberry consumption on SBP**						
Overall effect	12	0.96 (-0.26, 2.20)	0.12	0.13	31.7%	
Country						
USA	10	1.36 (0.41, 2.31)	0.005	0.02	79.4%	0.29
None-USA	2	-4.49 (-15.39, 6.40)	0.41	0.42	1.6%	
Study type						
Parallel	6	0.11 (-3.01, 3.24)	0.94	0.19	31.5%	0.54
Cross over	6	1.17 -0.14, 2.48)	0.08	0.14	39.6%	
Trial duration (week)						
<8	6	1.17 (-0.14, 2.48)	0.08	0.14	39.6%	0.54
≥8	6	0.11 (-3.01, 3.24)	0.94	0.19	31.5%	
Intervention type						
Fresh strawberry fruit	1	0.30(-3.46, 4.06)	0.87	-	-	0.73
freeze-dried strawberry powder	11	0.99 (-0.35, 2.35)	0.14	0.10	37.0%	
FDS powder dose (g/day)						
≤25	4	1.59 (0.21, 2.97)	0.02	0.67	0.0%	0.43
>25	7	0.49 (-1.88, 2.87)	0.68	0.03	56.9%	
Sex						
Both sexes	10	1.03 (-0.36, 2.42)	0.14	0.08	41.6%	0.61
					
Female	2	0.000 (-3.71, 3.71)	1.000	0.59	0.0%	
Age						
X<50	5	2.04 (0.87, 3.21)	0.001	0.99	0.0%	0.07
X≥50	7	-0.13 (-2.18, 1.92)	0.90	0.08	46.0%	
Baseline BMI (kg/m^2^)						
Overweight (25-29.9)	4	0.94 (-1.34, 3.24)	0.41	0.04	62.0%	0.77
Obesity (>30)	8	0.55 (-0.86, 1.97)	0.44	0.44	0.0%	
**Subgroup analyses of strawberry consumption on DBP**						
Overall effect	12	-0.33 (-1.31, 0.65)	0.50	10	36.0%	
Country						
USA	10	-0.02 (-1.06, 1.01)	0.96	0.17	29.4%	0.24
None-USA	2	-1.66 (-4.23, 0.90)	0.20	0.15	50.7%	
Study type						
Parallel	6	-0.73 (-2.43, 0.97)	0.40	0.24	24.6%	0.57
Cross over	6	-0.12 (-1.38, 1.13)	0.84	0.08	49.1%	
Trial duration (week)						
<8	6	-0.12 (-1.38, 1.13	0.84	0.08	49.1%	0.57
≥8	6	-0.73 (-2.43, 0.97)	0.40	0.24	24.6%	
Intervention type						
Fresh strawberry fruit	1	-0.50 (-2.74, 1.74)	0.66	-	-	0.88
freeze-dried strawberry powder	11	-0.32(-1.42, 0.78)	0.56	0.07	41.6%	
FDS powder dose (g/day)						
≤25	4	-0.32 (-2.05, 1.41)	0.71	0.11	50.3%	0.99
>25	7	-0.31 (-1.93, 1.29)	0.70	0.08	45.8%	
Sex						
Both sexes	10	-0.24 (-1.35, 0.86)	0.66	0.06	44.3%	0.58
Female	2	-1.00 (-3.47, 1.47)	0.42	0.42	0.0%	
Age						
X<50	5	0.96 (-0.11, 2.04)	0.08	0.85	0.0%	0.004
X≥50	7	-1.29 (-2.40, -0.18)	0.02	0.33	12.9%	
Baseline BMI (kg/m^2^)						
Overweight (25-29.9)	4	-0.12 (-1.74, 1.49)	0.88	0.06	58.4%	0.63
Obesity (>30)	8	-0.61 (-1.87, 0.64)	0.33	0.28	18.7%	

Abbreviations: CI, confidence interval; WMD, weighted mean differences; RCT, randomized clinical trial; R, randomized; C, Control; P, Placebo; DB, Double-blind SBP, systolic blood pressure; DBP, diastolic blood pressure; T2DM, Type 2 diabetes mellitus; MetS, Metabolic syndrome; HTN, Hypertension; BMI, Body mass index; USA, United State America; FDS, freeze-dried strawberry.

### Publication bias and sensitivity analyses

**Figure 8:**
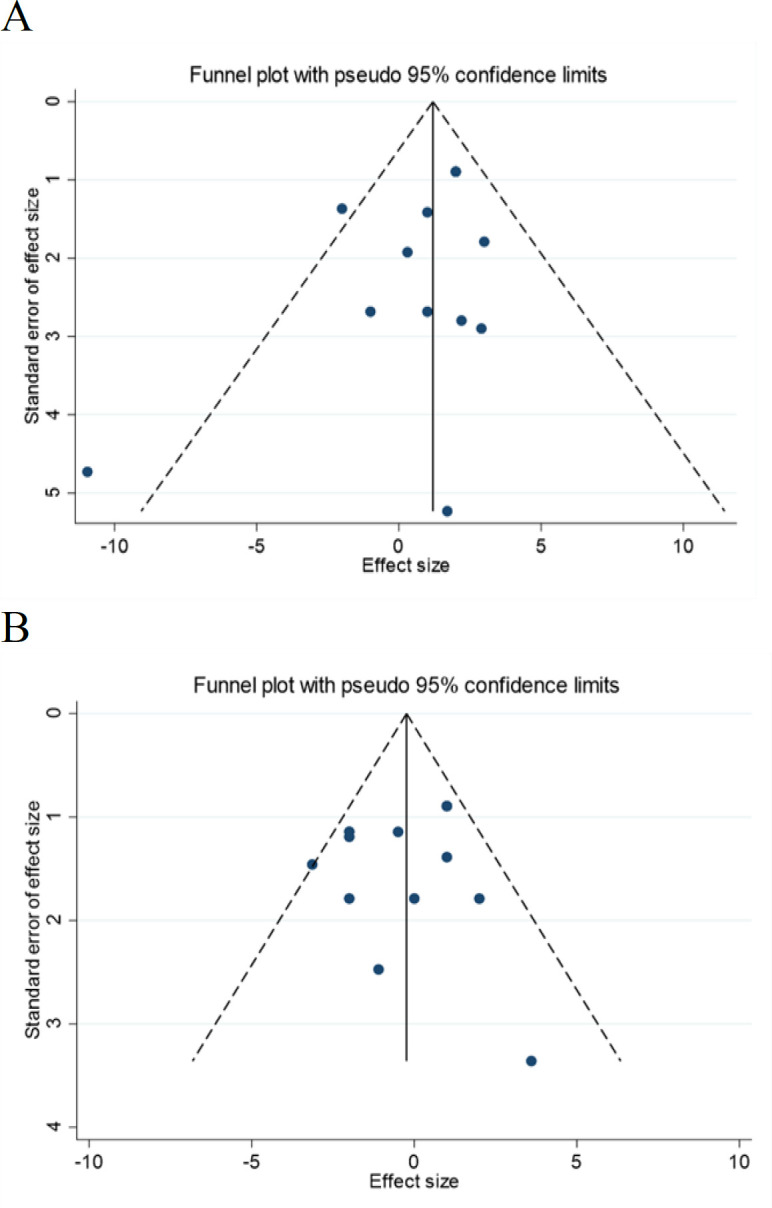
Funnel plots for the effect of strawberry consumption on (A) SBP (mmHg) and (B) DBP (mmHg)

The sensitivity analysis showed that the overall effect size for SBP was significantly changed after the omitting effect size of Amani et al. 2014 (WMD: 1.31 mmHg, 95% CI: 0.40 to 2.21) (Amani et al., 2014), or Basu et al. 2021(a) (WMD: 1.60 mmHg, 95% CI: 0.64 to 2.56) (Basu et al., 2021). However, the pooled effect size of DBP was not affected by the quality of one study. Begg correlation, Egger regression, and visual inspection of the funnel plots did not show any significant publication bias for the SBP (p_Begg_ = 0.37 and p_egger_= 0.22), and DBP (p_Begg_ = 0.94 and p_egger_= 0.79) (Figure 8A–B). 

### Grading of evidence

The GRADE guideline was used to evaluate the evidence's certainty (Guyatt et al., 2008). The quality of evidence on SBP and DBP was downgraded to moderate due to serious imprecision. Table 3 presents the GRADE profile.

## Discussion

*The current systematic review and meta-analysis of RCTs did not support the idea that strawberry consumption can help control blood pressure. In other words, the review's findings demonstrated that strawberry intake did not significantly alter SBP or DBP levels in comparison to the control groups. Also, it was demonstrated that a duration of 6 weeks for freeze-dried strawberry powder consumption is superior to other durations assessed in this review for reducing blood pressure. Although strawberry consumption generally had no significant effect on adult blood pressure, strawberry intake in individuals aged < 50 years or freeze-dried strawberry powder consumption at a dose of ≤ 25 g/day significantly increased SBP levels. However, a significant reduction in DBP levels was observed in individuals aged 50 or older following strawberry consumption. This may be related to the fact that SBP levels rise with ageing because of vascular stiffness or, in another explanation, a high prevalence of isolated systolic hypertension in people aged 50 or over (Pinto, 2007). Although this review did not indicate the relationship between strawberry intake and blood pressure level, there are three possible hypotheses to explain the potential effectiveness of strawberry consumption in lowering blood pressure. The first hypothesis suggests that strawberry intake may reduce blood pressure by lowering pulse wave velocity (PWV), the gold standard for measuring vascular stiffness. However, a study by Richter et al. in 2017 showed that strawberry intake did not significantly change the PWV levels compared to the control groups (Richter et al., 2017).* *The second hypothesis is that strawberries' blood pressure-lowering effect is mediated by modifying endothelial function and affecting the release of relaxing and vasoconstricting molecules such as nitric oxide (NO) and endothelin 1 (ET-1), respectively (Carretero and Oparil, 2000). However, a study by Feresin et al. 2017 showed that consumption of freeze-dried strawberries with doses of 25 g/day or 50 g/day for eight weeks could not significantly affect NO or ET-1 levels (Feresin et al., 2017).* *The third hypothesis suggests that intake of strawberries may prevent the damage of oxidants to endothelium by increasing the levels of superoxide dismutase (SOD) as an enzyme with antioxidant properties (Feresin et al., 2017). However, the study by Feresin et al. showed that consuming strawberries with a low dose (25 g/day) or high dose (50 g/day) did not change SOD levels compared to the control groups (Feresin et al., 2017). While this meta-analysis did not support the idea that strawberry intake can reduce blood pressure, strawberries have some compounds, such as polyphenols and flavonoids, which have been proven to lower blood pressure in some previous studies. *

**Table 3:** GRADE profile of Strawberry consumption for blood pressure in adults.

**Quality assessment**						**Quality of evidence**
**Outcomes**	**Risk of bias**	**Inconsistency**	**Indirectness**	**Imprecision**	**Publication Bias**	
SBP	No serious limitations	No serious limitations	No serious limitations	serious limitations	No serious limitations	⊕⊕⊕◯ Moderate
DBP	No serious limitations	No serious limitations	No serious limitations	serious limitations	No serious limitations	⊕⊕⊕◯ Moderate

CI: confidence interval; MD: mean difference

A meta-analysis conducted by Marx et al. in 2017 showed that intervention with polyphenol-containing compounds significantly improved SBP and DBP levels (Marx et al., 2017). A study by Macready et al. reported that consuming more than 4 servings of vegetables and fruits rich in flavonoids increased levels of NO metabolites in individuals at risk of CVDs (Macready et al., 2014). However, A meta-analysis carried out by Ellwood et al. in 2019 demonstrated that the consumption of flavonoid-rich fruits had no significant impact on SBP and DBP (Ellwood et al., 2019). In addition, the meta-analysis conducted by Daneshzad et al. in 2019 (Daneshzad et al., 2019) showed that anthocyanin supplementation did not have a significant effect on blood pressure.

### Adverse events

The eligible trials did not report any serious side effects of strawberry consumption. However, some included studies reported minor adverse events. The most frequent adverse events noted in the included trials were gastrointestinal complaints. In addition, skin and eye itching and headache were other complaints that were reported. Furthermore, strawberry intake in two studies was without adverse events (Basu et al., 2010; Basu et al., 2014), while in 3 studies, the incidence of side effects was not investigated (Jenkins et al., 2008; Feresin et al., 2017; Huang et al., 2021). 

To the best of our knowledge, this is the first GRADE-assessed systematic review and dose-response meta-analysis of data from RCTs investigating the effect of strawberry consumption on blood pressure. Another strength of our study is that the overall effect sizes of SBP and DBP were not dependent on just one particular pooled effect size. In addition, there was no significant heterogeneity and publication bias among the included effect sizes for SBP and DBP. However, this review included some limitations, such as a limited number of included trials. In addition, the quality of evidence for SBP and DBP was downgraded to moderate due to serious Imprecision.

 Our findings revealed that strawberry consumption did not lead to significant changes in SBP or DBP levels compared to the control groups. Subgroup analysis revealed that strawberry intake in individuals aged 50 or older significantly decreased DBP levels. However, consumption of freeze-dried strawberry powder at a dose of ≤ 25 g/day or strawberry intake in individuals aged less than 50 led to a significant increase in SBP levels. Furthermore, it seems that the consumption of freeze-dried strawberry powder for 6 weeks had the most optimal blood pressure-reducing effect compared to the duration of the other included studies. It is recommended that more RCTs investigate the impact of strawberry intake on blood pressure at higher dosages and larger sample sizes in the future to draw a definite conclusion.

## Data Availability

This published article contains all of the generated or analyzed data obtained during this investigation.
